# Iodipin

**Published:** 1907-07-06

**Authors:** 


### POINTS IN TREATMENT.

### IODIPIN.

NOBODY has the least doubt of the efficacy of potassium iodide in the relief of tertiary syphilitic ulcerations, gummata, and sores; especially if the patient has been through a course of mercurial treatment at a former date, or is given mercury at the same time as the potassium iodide. When mercury has not been previously given, and the potassium iodide is tried alone, the results are much less good in many cases; the reason for this appears to be that mercury, when once given, remains stored for many years in the body, more especially in the bones, so that the administration of iodides to persons who have had mercurial treatment leads to the setting free of some of this as soluble iodide of mercury.

There are, of course, many syphilitic affections which are not influenced by drugs at all. Locomotor ataxy, for example, in which the sclerosis of the posterior columns of the cord, though primarily due to syphilis, is ultimately identical with what would follow section of the columns with a knife. Potassium iodide and mercury would not cure the sclerosis which would result from section of these columns: they will have no more effect upon the sclerosis because it happens to be due to syphilis instead of a knife. The iodides only cure the granulomatous results of syphilis; they lead to cure of gummata by conversion of the small round celled collections into mature fibrous tissue, the result being a healed scar; but they will not remove the scar itself, nor the results of the scar, should there be any, as in the case of gummatous meningitis and so on.

If potassium iodide could always be well borne by every syphilitic patient there would be no need to search for any other means of administering an iodide; hence nobody is likely to try anything but iodide of potassium in these cases in the first instance. Unfortunately, however, there are many patients who suffer acutely from the effects of the drug itself, the three chief troubles arising from it being acne boils, extreme mental and bodily depression, and loss of flesh. The depression is the most serious of the three, but not infrequently all three occur together. Before giving up the iodide of potassium, the physician will probably make several changes in his prescription to try and circumvent its ill effects. In the first place, he will no doubt follow the old adage: "If potassium iodide produces acne or depression, double the dose, and the trouble will often cease." He may find that whereas five-grain doses are not enough to cure the syphilitic trouble, and whereas ten-grain doses, though curing the syphilis, are unbearable to the patient, 20 to 30-grain doses three times a day may be well borne, and may effect a rapid cure. If this be not so, he will probably prescribe 3-minim doses of liquor arsenicalis with each dose of the iodide, and thereby lessen the acneiform eruption; or he may prescribe 15 minims of aromatic spirits of ammonia in each dose to lessen the depression.

Supposing, however, that none of these measures succeed, the next step will probably be to prescribe part of the iodide as another salt than that of potassium. Unfortunately, sodium iodide, ammonium iodide, and lithium iodide will none of them produce the rapid cure of gummata that potassium iodide will; hence it is not possible, as a rule, to eliminate all the potassium iodide from the prescription. Nevertheless, part of the iodide can be given as the sodium or ammonium salts, provided more of the latter are ordered than is equivalent to the potassium iodide replaced. For example, if the patient should require 15-grain doses of potassium iodide, but cannot take more than ten grains at a time without suffering from severe depression, it is possible to obtain a good effect equivalent to that of 15 grains of potassium iodide by giving ten grains each of sodium, ammonium, and potassium iodides, thus:—

| | | |
|---|---|---|
| ℞ | Potassii iodidi ... ... ... ... | gr. x. |
| | Sodii iodidi ... ... ... ... | gr. x. |
| | Ammonii iodidi ... ... ... ... | gr. x. |
| | Spiritus ammonii aromatici ... ... | ♏xv. |
| | Aquam chloroformi ad ... ... ... | ℥j. t.d.s. |

Notwithstanding attempts such as the above to obviate the depressing effects of potassium iodide, there will still be patients who absolutely cannot take potassium iodide in the needful quantities at all. It becomes necessary to find some other iodine preparation that they can take. There are many to be bought, in the form of syrups, hydriodic acid, and so on; but it is always difficult to know which of the advertised remedies is any use, and which useless. Every advertised article is naturally regarded with suspicion, but it is wrong to suppose that all are to be avoided. The profession must be grateful to its members who try to weed out the bad preparations from the good, and when the good are known they will be used in appropriate cases. As an example of an advertised medicine which is proved to be of immense benefit in some conditions, we have aceto-salicylic acid (=aspirin); as an example of an iodide which produces all the good without many of the bad effects of potassium iodide we have iodipin. Many medical men of standing have tried it, and have reported well of its effects. It will probably not be tried until potassium iodide has been found impossible of use in any particular case; but, failing potassium iodide, it is good to know that iodipin can take its place.

The preparation is a combination of iodine with sesame oil, discovered by Winternitz; it can be prescribed in various strengths, the two most usual being a 10-per cent. solution and a 25-per cent. solution. Upon the continent it is largely administered by subcutaneous or intramuscular injection; if it could be used only by this method it would commend itself to few, but, among others, Dr. Stopford Taylor and Dr. MacKenna, of the Liverpool Skin Hospital, have watched the results of giving it by the mouth, and they find them excellent. Some very severe cases of tertiary syphilis were thus treated by them, with rapid improvement in the condition. They prescribed 30 minims of 25-per cent. iodipin in milk, three times a day, about two hours after food. In twelve days, after taking 1½ oz. of iodipin altogether, the lesions, previously very severe, were upon the high road to being healed.

They find that whereas potassium iodide is very rapidly eliminated from the body, particularly in the urine, iodipin is thus lost much more slowly; even two months after the last dose iodine has still been found in the urine. This slow elimination is possibly one of the chief causes of its efficacy; in any case, no symptoms of iodism, and no depression is observed, and the patients gain, rather than lose flesh. We wish that iodipin were a pharmaceopœial and not a proprietary drug, for it is a drug which will almost certainly prove useful in many cases where potassium iodide cannot be borne.

